# Digging through the (Statistical) Dirt: A Reproducible Method for Single-Molecule Flicker Noise Analysis

**DOI:** 10.1021/acs.jpcc.4c07780

**Published:** 2025-02-14

**Authors:** James M. F. Morris, Jarred Potter, Demetris Bates, Chuanli Wu, Craig M. Robertson, Simon J. Higgins, Richard J. Nichols, Paul J. Low, Andrea Vezzoli

**Affiliations:** a Department of Chemistry, University of Liverpool, Crown Street, Liverpool L69 7ZD, United Kingdom; b Stephenson Institute for Renewable Energy, Peach Street, Liverpool L69 7DF, United Kingdom; c School of Molecular Sciences, University of Western Australia, Crawley, Western Australia 6009, Australia

## Abstract

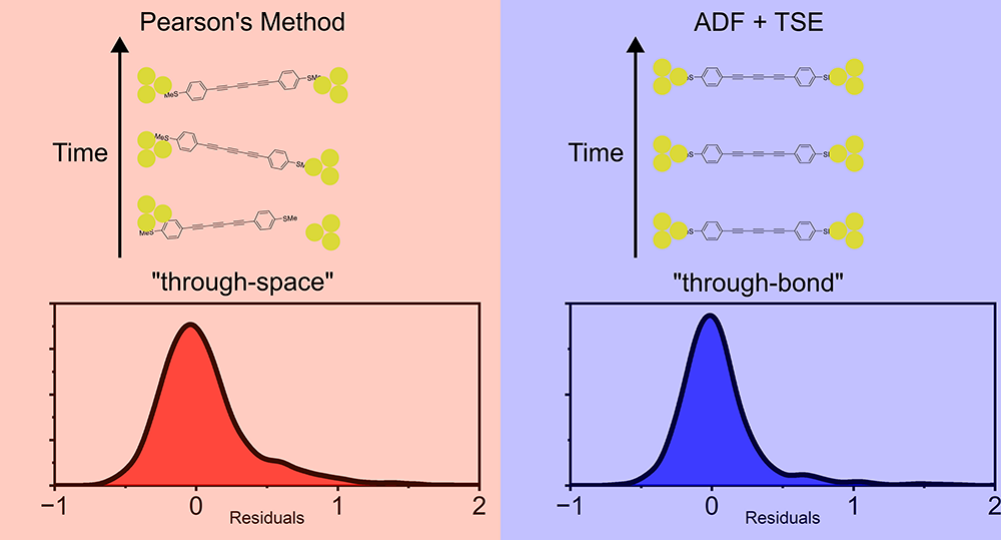

Flicker noise analysis has found widespread use in the molecular electronics community over the past 9 years. The noise power of the junctions and the value of its scaling exponent *n* provide information on the spatial overlap of scattering states in single-molecule junctions and give unique insights into quantum transport phenomena at the single-molecule level. The predominant drawback of this analytical tool is the inconsistency of the methodologies employed, resulting in irreproducibility across data sets acquired in different conditions or in different laboratories. Herein, we provide a pathway to a more reproducible methodology, detailing issues with the currently accepted correlation techniques employed and introducing the use of more statistically robust data processing criteria and lower thresholds for data acquisition parameters.

## 1 Introduction

Over the last few decades several techniques for the fabrication^1,2^ and characterization of single-molecule junctions, *i.e.*, nanoelectronic devices in which a single molecule is contacted between two biased (often metallic) electrodes, have been developed, with methods that probe their physical properties going significantly beyond the simple determination of their charge transport efficiency. Techniques have arisen that allow accurate experimental determination of the dominant transport resonance position,^3,4^ the electronic coupling to the electrodes,^3,4^ and even the evaluation of mechanisms and pathways of coherent tunneling charge transport across single-molecules. The latter, representing the ability to analyze the pink (“flicker”) noise signature of individual molecular junctions to discern *through-bond* or *through-space* transport is of fundamental importance to the molecular electronics community,^5^ and it has proven to be an effective tool as evidenced by the acceleration of its use over the last 9 years.^6^

The method, an evolution of the noise analysis originally used to characterize atomic point contacts,^7−9^ involves integrating the conductance power spectrum (power spectral density, PSD) over a bandwidth of 100–1000 *Hz* to obtain the noise power (*NP*). The arbitrary limits of integration were chosen to reduce the impact of mechanical and thermal noise^10^ which may dominate, respectively, at *f* < 100 Hz and *f* > 1 kHz. It was found that the relationship between *NP* and the average conductance (*G*_*AVG*_) followed a power law
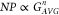
1with *n* typically having a value between 1 and 2. For values of *n* closer to 1, the system is considered to be *through-bond* coupled (*i.e.*, there is no break in the spatial overlap of the electrode-molecule-electrode wave functions), while as *n* approaches 2 it becomes more *through-space* coupled (*i.e.*, there is a break in the overlap of the electrode-molecule-electrode wave functions). Since the demonstration of this analytical technique and its original use as a way to probe the molecule-electrode interface,^6^ it has been applied to the validation of quantum interference (QI) effects,^11,12^ the characterization of sigma-conjugated insulating molecules,^13^ as a tool to monitor junction evolution under continuous pulling,^14^ and to probe other phenomena that may interrupt the spatial electron density of the molecule-assisted scattering states across the whole junction.^15−18^ As such, it has also found use in determining the formation of dimeric junctions,^19−21^ where two molecules bridge the nanogap held together through nonbonding π–π interactions (*e.g.*, π*-*stacking).

However, with the increasing use of a technique comes the need for standardization, to ensure reproducibility and reliability of the experimental results. Using different equipment, data acquisition protocols, and data processing methodologies leads to different values of *n* obtained for the same molecular wire,^6,18^ and our findings show that *n* is likely prone to overestimation, especially if data sets are flawed by low statistics, nonstationarity, or if a Pearson’s correlation method is used on data in the presence of outliers or skewed data sets. In this contribution, we describe some of the challenges faced by researchers using this technique and rationalize the choices made at each step in the analysis process, proposing a standardized method for flicker noise analysis that yields more robust and reliable results. We demonstrate how the use of a Thiel-Sen estimator coupled with an Augmented Dickey-Fuller stationarity test and sensible data trimming ensure better accuracy and reproducibility, and we propose a lower threshold for data acquisition speed and measurement bandwidth (*e.g.*, number of individual junctions fabricated) that allows the calculation of accurate and statistically robust values of *n.*

## 2 Methods

### 2.1 Materials

The syntheses of molecules **1**(15) and **2**(44) used in this study are reported elsewhere. Chemicals used in the scanning tunneling microscope–break-junction measurements (STM-BJ)^2^ were purchased from TCI UK and used without further purification.

### 2.2 Single-Molecule Charge Transport Characterization

We used a modified scanning tunneling microscope (Keysight 5500 SPM) to fabricate single-molecule junctions using the break-junction method.^2^ In brief, after regular approach of the STM Au tip to a freshly evaporated Au-on-mica substrate under constant DC bias, the feedback loop is disconnected and the voltage to the piezoelectric transducer is controlled by an Arbitrary Waveform Generator (AWG, Keysight 335522B) by applying a voltage signal to the piezoelectric transducer. The tip is first driven into the substrate, thus creating a metallic contact with conductance significantly greater than the quantum of conductance *G*_0_ ≅ 77.48 μS, and then withdrawn, either at constant speed or using a chosen ramp (*e.g.,* a step function). The process is performed in a solution (1 *mM*, 1,3,5-trimethylbenzene) of the molecular wire of interest. During the withdrawal process, the metallic contact is thinned and ruptured, and molecules provided with appropriate metallophilic termini can self-assemble in the freshly created nanogap, bridging the space between the two electrodes and fabricating a single-molecule junction. The withdrawal process is continued until the junction is broken, and the tip is driven again into the substrate to generate a fresh metallic contact. Data are continuously acquired through a PXI system (National Instruments PXIe-1062Q/PXIe-4464/PXIe-PCIe8381). A detailed description of the instrument can be found elsewhere.^45,46^ Instrument control, data acquisition and data analysis are performed with custom LabVIEW VIs. STM-BJ experiments were carried out at a sampling rate of 20 kHz for all molecules and flicker noise measurements were performed at 100 kHz and 40 kHz for molecules **1** and **2** respectively with the piezo ramp detailed in section 3.3 below. During all measurements, the tip was occasionally, albeit rarely, moved to another area of the substrate to maximize the number of measurements that could be used for further analysis. As such, junction formation probabilities should not be drawn from the data reported here.

## 3 Results and Discussion

**Figure 1:**
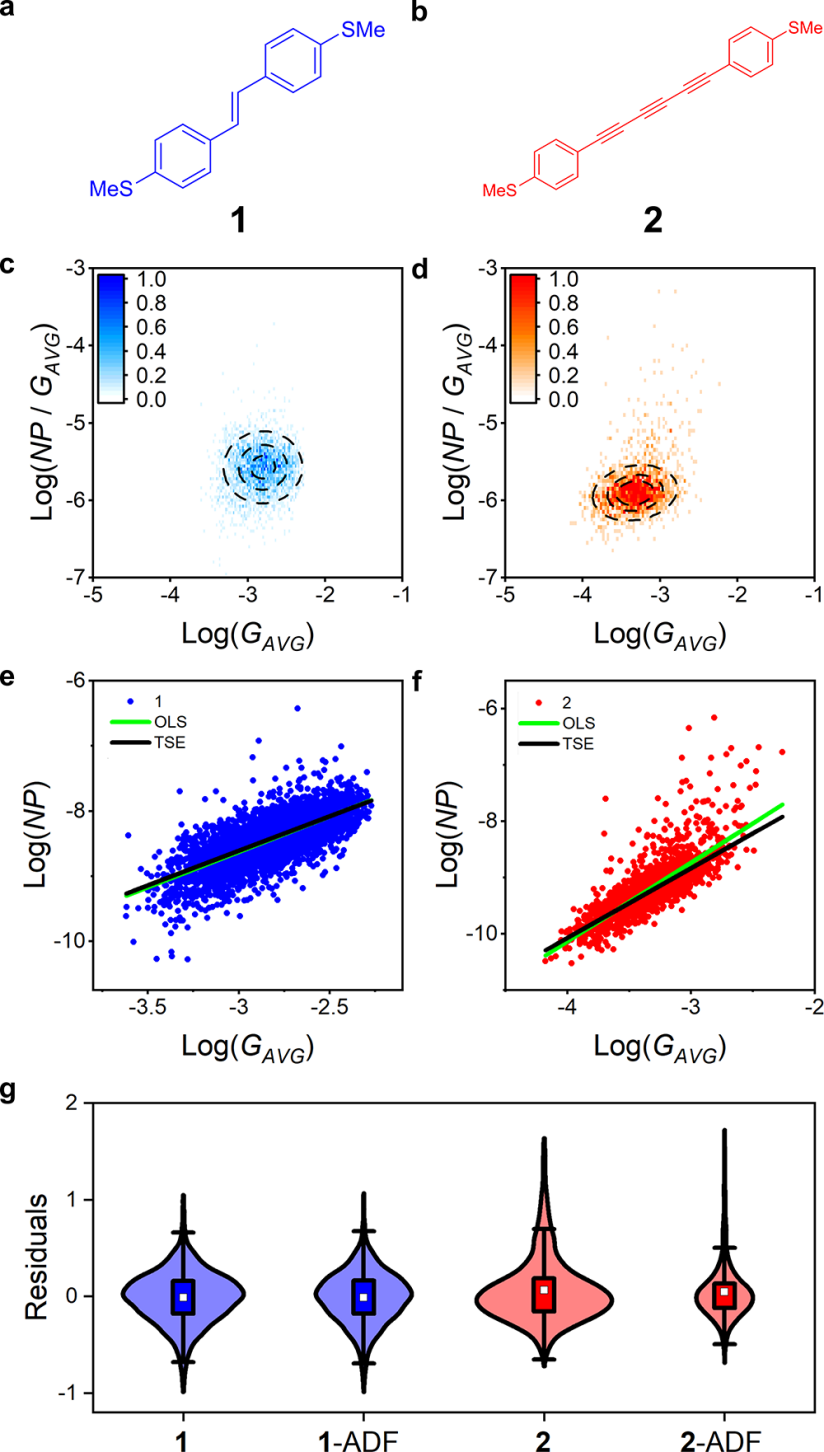
Structures of compounds **(a) 1** and **(b) 2**. The normalized flicker noise power vs *G*_*AVG*_ density maps of **(c) 1** (3125 traces after selection) and **(d) 2** (1843 traces after selection). Scatter plots for **(e) 1** and **(f) 2** after applying the augmented Dickey-Fuller (ADF) test with the ordinary least-squares (green) and Theil-Sen estimator (black) fits overlaid. **g)** Violin plots for the residuals of the fit for **1** and **2** with and without the ADF test applied; white squares represent the mean, the boxes form the interquartile range (IQR) and the whiskers are at 1.5 IQR.

We focused on two molecular wires (**1** and **2** in Figures 1a and 1b respectively) when testing the parameters for the flicker noise methodology. We chose these compounds for our investigation as the value of *n* for **1** has been reported by three independent studies^6,15,18^ as being close to unity, thus suggesting a high level of *“through-bond”* coupling with high junction stability owing to the consistency in results across different research groups. Compound **2** was selected not only because of its archetypal molecular wire-structure arising from the linear, π-conjugated oligoynyl (-{C≡C-}_*x*_) backbone, but also due to its higher junction instability relative to **1**. Details on an oligophenylene ethynylene (OPE) derivative (molecule **3**) can also be found in section S1.3 of the SI.

In both cases, we used the scanning tunneling microscopy break-junction method^2^ to fabricate and characterize electrical transport through single-molecule junctions. In this method, Au point contacts (having conductance *G* equal to the conductance quantum *G*_0_ ≅ 77.485 μS) are repeatedly fabricated and ruptured under a DC voltage by driving an atomically sharp Au tip into and out of contact with an Au substrate. Performing the process in a solution of the desired molecular wire allows single-molecule junctions to spontaneously self-assemble in the nanogap left after rupture of the atomic contact. Their formation, evolution and eventual rupture is controlled by the signal applied to the piezoelectric transducer responsible for moving the tip respective to the substrate, and the whole process is followed in real-time by recording the conductance of the junction as a function of the applied piezo signal. When measurements are to be performed in the domain of time, such as for flicker noise measurements, then the movement of the piezo is stopped once the junction is in place, and current is recorded as a time-series. The process is performed thousands of times, and the data is processed to deliver statistical distributions of the property to be measured (*e.g.*, conductance, noise power, etc.).

### 3.1 Calculation of the Value of *n*

As discussed in the introduction, the key result of flicker noise analysis is the scaling exponent of the power law existing between the *NP* and *G*_*AVG*_ of a single-molecule junction (eq 1). Following initial attempts at obtaining *n* by graphical means, by plotting a *NP*/*G*_*AVG*_^*n*^ vs *G*_*AVG*_ heatmap and fitting it to a bivariate distribution^6,15^ over a range of values of *n*, a direct Pearson’s correlation method was introduced.^19^ This method was based on the minimization of the absolute value of Pearson’s correlation coefficient, *r*, between *NP*/*G*_*AVG*_^*n*^ vs *G*_*AVG*_ by iteratively changing the value of *n* until *r* = 0.^14,19^ This has the drawback of introducing issues with the accuracy of *n* due to the nonlinearity of the data set. Alternatively, the value of *n* can be obtained from the slope of the *Log*(*NP*) and *Log*(*G*_*AVG*_) plot. There are a variety of methods to evaluate this, the most common being ordinary least-squares (OLS) which results in the value of *n* that minimizes |*r*| between the linearized data set of *Log*(*NP*/*G*_*AVG*_^*n*^) vs *Log*(*G*_*AVG*_) (see section S2.1 of the SI for details). This method is equivalent to more recent attempts at evaluating *n* on the linearized data set.^18^ One of the major advantages of the OLS method over other techniques lies in a greatly simplified calculation of the central value and standard error of *n*.

While still the method of choice and the most prevalent in the literature for the direct calculation of *n* from noise power data,^19,22^ Pearson’s-based methods are highly sensitive to outliers and are inefficient at handling long-tailed distributions.^23−27^ Due to the inherent instability of the STM-BJ setup and the data processing criteria used to isolate the contributions of molecular junctions from that of tunnel junctions,^6,15^ outliers are a distinct possibility and a particular error distribution cannot be assumed.^28^ These issues effectively make a Pearson’s-based approach biased and lacking in statistical robustness.

A more robust, unbiased, estimate of the slope, *n*, is given by the Theil-Sen estimator (TSE)^27,29^ (see section S2.3).^23^ This method involves evaluating the median of the slopes between all possible values of *Log*(*NP*) and *Log*(*G*_*AVG*_).^29,30^ Therefore, it can be represented by
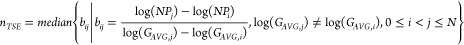
2

The influence of outliers and long-tailed, skewed error distributions on the final value of *n* calculated with the two methods can be readily observed in Figure 1. As a control experiment, we can observe how in **1** there is almost no difference between the OLS and TSE results due to the absence of a significant outlier population (Figure 1e). The greater number of outlying data points of the data set for **2** (Figure 1f) biases the OLS result upward compared with the TSE. Historically, the primary drawback of the TSE method when compared to OLS was its relative difficulty of computation.^31^ However, with currently readily available computing (*e.g.*, the Intel i5 4-core 3.2 GHz, 8 GB RAM used in this study) the value of the slope can be obtained within time scales on the order of seconds. While other median-based regression methods may also be used in place of the TSE, we advocate for using the TSE because it is a relatively robust median regression method, with a breakdown point of 29%, without being overly insensitive to outlying data (*e.g.*, the repeated median regression).^23^ It also parallels OLS as, while OLS minimizes the absolute value of Pearson’s *r*, the TSE minimizes the absolute value of Kendall’s rank correlation coefficient, *t*.^26,29^ The errors associated with this slope can also be evaluated from an analytical expression for the confidence intervals and are corroborated by bootstrapping with replacement (see section S2.4).^23,29,32^ Furthermore, the estimator has other desirable properties such as asymptotic normality and its ability to handle stochastic regressors such as those found in break-junction data set.^23,29,33^

### 3.2 The Importance of Stationarity in the Conductance Time-Series Data

The conductance of a stable, equilibrated molecular junction at a fixed displacement, once formed, should not change beyond random fluctuations.^34^ However, when using scanning tunneling microscopy methods, after formation and stabilization of a single-molecule junction the piezoelectric transducer responsible for keeping the nanoelectrodes at a specified distance may suffer from creep or thermal drift, causing premature rupture. These changes may also result in junction stretching, junction compression, or changes in binding configuration (*e.g.*, lateral coupling,^35^ a form of enhanced Au-π interaction).^36^ Under these circumstances, the wave function overlap from one electrode to the other changes in a time-dependent way. As such, the noise and *G*_*AVG*_ change with time and therefore, the results from flicker noise analysis are uninterpretable.

A resolution to this problem, and one which is standard in time-series analysis procedures, is to employ a stationarity test on the data set. This ensures that the *statistical* properties of the conductance trace do not change with time. Among the various methods available, we used the augmented Dickey-Fuller (ADF) hypothesis test, at a significance level of α = 0.05 (to balance type 1 and type 2 errors). If the null hypothesis was rejected, we retained the junction and assumed it was sufficiently stationary for further analysis (further details on the ADF test are provided in the SI, section S1.3). Other methods to evaluate stationarity such as the Kwiatkowski Phillips-Schmidt-Shin (KPSS) test could be used, but as KPSS rejects stationarity not nonstationarity (as with ADF), we elected to use the ADF test. However, for a stricter measure of stationarity both could be used as is often the case when performing autoregressive fractionally integrated moving average (ARFIMA) analysis.

Furthermore, the effect of employing the ADF test can be seen in Figure 1g to reduce the heavy-tailed nature of the residuals. In the case of **1**, this has little effect on the overall distribution, but for **2** it can be seen to effectively de-emphasize the skewed tail. While data acquisition and analysis routines were consistent for both compounds (see methods), it is evident from the violin plots in Figure 1g that the data set obtained from **2** shows greater skew in the residuals, likely due to time dependent variations in the conductance traces.

We hypothesized that inclusion of the ADF test would increase the reproducibility between data sets of the same molecule and make the comparison between two molecules possible by ensuring that the statistical parameters were temporally constant. To verify this, we determined the value of *n* with and without the stationarity test and it can be seen in Table 1 that both inclusion of the ADF test and use of the TSE has a greater effect on **2** than **1**, indicating that **1** did indeed form more stable junctions that were comparably more stationary with respect to **2**.

**Table 1:** Results of the Scaling Exponent *n* of **1** and **2**, Calculated with OLS without the ADF Test (*n*_*OLS*_), OLS with the ADF Test (*n*_*OLS*+*ADF*_), TSE (*n*_*TSE*_) without the ADF Test and TSE with ADF (*n*_*TSE*+*ADF*_)

**molecule**	***n*_*OLS*_**	***n*_*OLS+ADF*_**	***n*_*TSE*_**	***n*_*TSE+ADF*_**
**1**	1.08 ± 0.02	1.09 ± 0.02	1.05 ± 0.02	1.06 ± 0.02
**2**	1.63 ± 0.02	1.40 ± 0.03	1.45 ± 0.01	1.24 ± 0.02

Our data therefore suggests that the reproducibility across independent measurements observed for **1** arises from its inherent stationarity. This is because the junction stability of **1** results in few outliers being present in the corresponding data set and OLS returns the same value of *n* as TSE. On the other hand, junctions formed from **2** suffer from greater instability and nonstationarity, giving rise to the long, heavy-tailed residuals that can be observed in Figure 1g. Given that stationarity is a *conditio sine qua non* for time-series analyses and that junction stationarity is strongly influenced by experimental parameters (*e.g.*, quality of the piezoelectric transducers, open-loop or closed-loop operation, *etc.)* and varies from molecule to molecule, or in extreme cases even from measurement to measurement, our data shows that a stationarity filtering procedure reduces the effect of these external variables on the value of *n*.

The scaling exponent obtained from the OLS fitting would lead to a description of the junction formed from **2** involving less through-bond coupling, when in reality the value obtained from this method of analysis reflects the mechanical instability of the junction, and the greater data spread. The data set is better analyzed using the methods proposed here, which gives values much more consistent with the through-bond coupling mechanisms associated with the thioanisole anchor groups and the chemical nature of the molecule, albeit it increased from the idealized value *n* = 1.

### 3.3 Acquisition Speed Requirements

**Figure 2:**
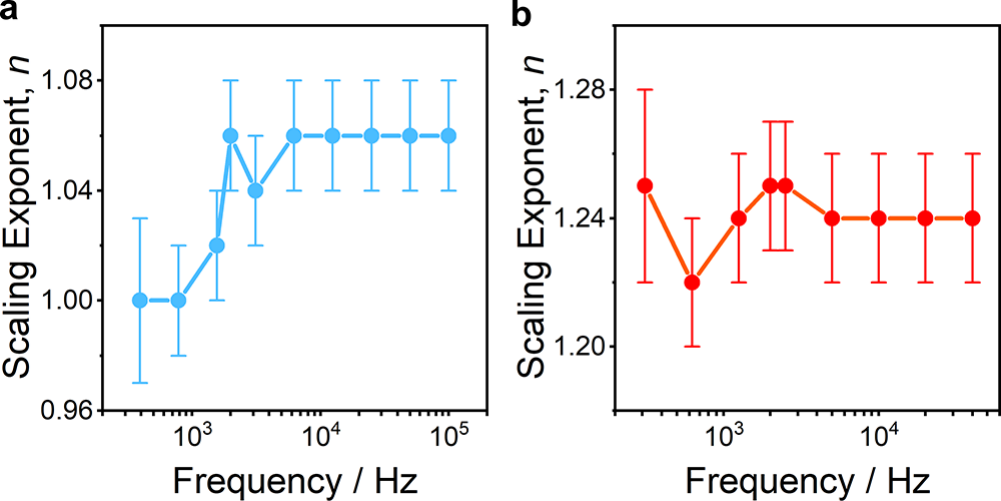
Scaling exponent *n* vs sampling frequency for **(a) 1** and **(b) 2** along with their respective standard errors. Reduced sampling was obtained by down sampling the original data sets.

The Nyquist-Shannon sampling theorem states that the sampling frequency of a time series should be at least twice as large as the signal’s bandwidth, *B*.^37^ Even though we integrate only up to 1000 Hz, this cannot be chosen as the true bandwidth as *B* is likely infinite given that we are investigating the signal noise (see section S3 of the SI for details). Although previous sampling rates have varied from 10–100 kHz,^6,18,38−40^ the choice of sampling frequency has not been previously explored for flicker noise analysis. There are indeed advantages to acquiring data at the lower bandwidths, as it lowers the computational cost of further processing and reduces the overall size of the data sets. Furthermore, given that the absolute noise floor of transimpedance current amplifiers (regularly used in STM-BJ measurements) is proportional to the square root of the bandwidth (), lower acquisition frequencies enable reliable measurements on molecules with less efficient charge transport. We investigated the effect of reduced sampling on the value of the scaling exponent in Figure 2 for **1** and **2** and found that, using the ADF test and TSE, data acquired at rates above ∼5 kHz begin to show self-consistency, while significant changes in the value of *n* were found prior to this threshold. Despite the consistency across molecules **1** and **2**, this result is nonexhaustive and lowering the sampling rate for new classes of molecule and molecule-electrode contacts would require further analysis of the sampling rate threshold for the particular system being studied. The revised methodology we propose therefore enables the acquisition of data at lower frequencies. However, we note that the accuracy of the integral of the power spectra depends on the number of data points being integrated. This value is related to the product of the sampling rate and the time period that the junction is held for. As such, shorter hold periods may require higher sampling rates to retain the accuracy of *n*.

### 3.4 Data Selection and Processing

In a typical STM-BJ experiment different geometries (either at the electrode-molecule interface or due to the presence of conformers, rotamers, or isomers) may result in multiple conductance peaks in the 1D conductance histograms. Therefore, 2D conductance displacement histograms are commonly used to distinguish each peak by its associated electrode separation to determine which peak most likely corresponds to the molecule sitting in the junctions in its most extended “ground” state and select it for further analysis. To achieve this, the STM tip is retracted to the distance between the anchoring groups on the molecule. However, when the electrodes are separated by a specified displacement it is not possible to determine with certainty if the nanogap is the size that was sent to the piezo transducers. This uncertainty is due to the stochastic rearrangement of the Au atoms on the apexes of the two electrodes upon rupture of the *G*_0_ atomic contact, which cause the size of the freshly formed nanogap to vary by >1 nm.^41^ As such, we employed a series of conditions to ensure that the selected junction traces were representative of the fully extended single-molecule junction.

**Figure 3:**
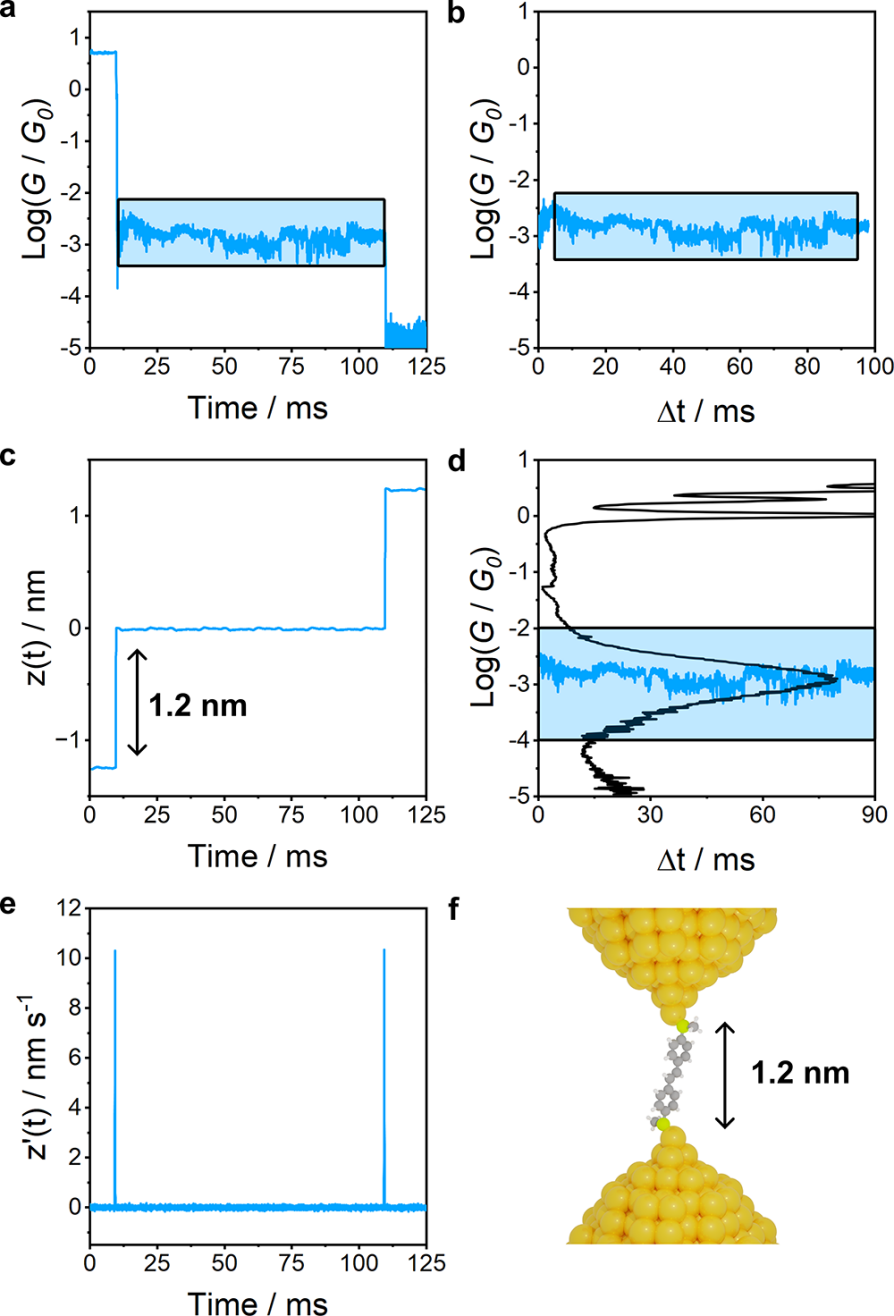
(**a)** Characteristic conductance trace, where the 100 ms hold period (blue square) is cut according to (c) the signal imposed to the piezoelectric transducer and (e) its derivative. (**b)** The junction is trimmed to a 90 ms region (blue square) and (d) it is compared with the linearly retracted STM-BJ conductance histogram. If its initial and final 5% fall within ±2σ of the Gaussian distribution the trace is saved for further processing. The retraction length in (c) corresponds to the electrode separation shown in (f). Data shown was collected using compound **1**.

After isolation of the conductance traces by way of the first derivative of the piezo signal, as shown in Figures 3a, 3c and 3e, the initial conductance values of the traces at the peak of the derivative were required to align with the *G*_0_ rupture commonly associated with the formation of sharp metallic contacts. If this condition was not met, the traces were removed. This ensured that the traces selected were associated with the full step of the electrode separation.

Figure 3c shows the step function piezo ramp that was used in this study. However, a slower retraction rate with a gradual rise to the plateau region is frequently used to reduce the effect that the piezo movement has on the noise.^6,14^ As reported by Pan et al.,^14^ faster retraction rates of the rising portion of these ramps lead to larger values of the scaling exponent. We therefore investigated the effect of using the most extreme case of the step function, where the retraction rate is limited only by the piezo response.

In such a case, we found that after 2 ms, the scaling exponent settles to a consistent value. Therefore, given this and the results mentioned earlier,^14^ we hypothesize that the increase in *n* from faster ramps is caused by a convolution of the piezo relaxation and the contact reorganization postretraction. To minimize these phenomena and improve reproducibility across laboratories where different procedures and equipment are employed, we propose trimming the first and last 5 ms of each single conductance trace when using a step function piezo ramp to ensure that only the portion of the junction with a relaxed piezo and stable molecular bridge is used for flicker noise analysis.

Given that slower ramps converge to a consistent value for *n*, it is possible that slower rates require less data trimming. For instance, the value of *n* for **1** (Table 1) which was obtained from the step function ramp after trimming, agrees with the result from the slower retraction used by Adak et al.^6^ Therefore, an analysis of the trimming requirements until convergence of the value of *n* should be performed when different ramp speeds are employed. We also trimmed the last 5 ms to avoid the inclusion of any piezo movement at the end of the hold period (*e.g*., artifacts induced by asynchronous acquisition of data across multiple channels), resulting in the isolated 90 ms as shown in Figures 3d and 3e.

Following this, the mean conductance of the first and last 5% of the isolated junctions were computed and those that fell within two standard deviations σ of the linearly ramped molecular junction conductance histogram peak were selected for further analysis. This was to ensure that the fixed height junctions were comparable to the metal-molecule-metal junctions formed in the STM-BJ measurements. The 5% sample size sets were chosen because the scaling exponent stabilizes above this value and the sampled 5% data sets were large enough for the mean to be stable. The use of a 2σ threshold minimizes data set filtering but it is only applicable when the preamplifier saturation region or its noise floor, along with any satellite conductance peaks associated with nondominant geometries, do not fall within the envelope. Selecting a smaller multiple of σ may be appropriate in the case of overlapping peaks or conductance features too close to the instrument noise floor, and it has been used in the literature with sensible results.^20^ However, since the use of a smaller range would have the effect of probing fewer junction geometries, the exact value used should always be reported for transparency, and consistency should be applied when different data sets are compared. Finally, prior to evaluation of the power spectral density, the average conductance of the trace was subtracted, thus centralizing each trace. This was done to remove any residual dependence of the noise power on *G*_*AVG*_ while avoiding the introduction of artificial ∼1/*f*^2^ type behavior into the PSD due to the presence of the DC component. The result of this is that the frequency dependence of the power spectra drops from the already reported flicker-type noise (1/*f*^1.4^)^6,42,43^ to true flicker noise (1/*f*).

### 3.5 Sample Size Relation to Accuracy and Precision

**Figure 4:**
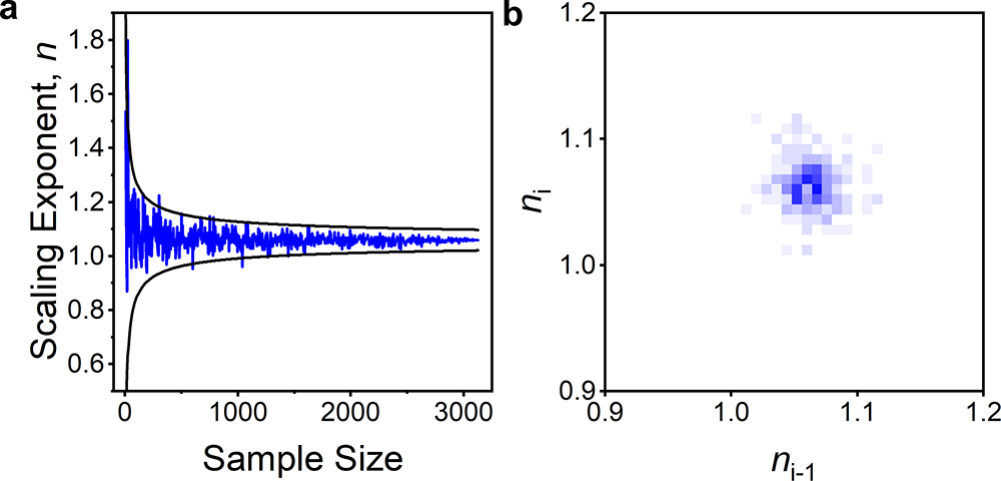
(**a)** The scaling exponent *n* for **1** plotted against the sample size. Black lines represent the standard error of *n* for the final data point scaled by , where *N* is the sample size of the final data point and *s* is the sample size of the current data point. (**b)** The autoregression lag-1 plot of (a), compiled with 125 bins/*n*.

The sample size (*i.e.*, the number of conductance vs time traces used for statistical analysis and correlation) after data selection is typically small due to the data selection requirements to ensure high quality stationary junctions. However, it can be seen from Figure 4a that, as one would expect, smaller sample sizes are representative of the larger sample sizes, but with varying errors. This independence of sample size and the zero autoregression slope in Figure 4b indicates that *n* is simply a random number centered around the population statistic. Therefore, there is no definitive sample size threshold that can be chosen below which accuracy becomes an issue, making this simply a matter of precision. Thus, reporting of the errors associated with the scaling exponent is, as expected, important and should be common practice.

## 4 Conclusions

Combining the many uses of flicker noise analysis along with its straightforward measurement, the technique has developed into an invaluable experimental tool. However, the method has been limited by inconsistencies in data processing and analysis, constraining its application to only qualitative analysis. In this contribution we have reviewed the methodology of flicker noise analysis and have presented a more careful protocol using robust statistical methods to obtain results that can be consistently and reliably interpreted. Using the Theil-Sen estimator in place of ordinary least-squares or other Pearson’s correlation-based approaches alongside a stationarity test such as the augmented Dickey-Fuller test excluded outlier or long-tailed error term influences without the necessity of arbitrary cut off regions, resulting in the analysis remaining robust in the face of potentially suboptimum junction isolation regimes and even different classes of molecules.

A sufficiently accurate and reproducible method provides the foundations for both comparisons between molecules measured in separate studies, and gauging what limitations the method may have. We have shown that with careful consideration of the analysis and processing procedures, flicker noise data can generate results that are *quantitatively* reproducible, making the method more reliable in the presence of unstable junctions and less dependent on instrumentation. This more quantitative method also provides a starting point for theoretical descriptions that could result in a more direct relation between the physical properties of the systems and ***n***.

## Data Availability

Raw STMBJ data for all species discussed in this contribution along with Theil-Sen estimator (with error evaluation) (LabVIEW) and augmented Dickey-Fuller processing codes (python) are available under a CC-BY license in the University of Liverpool Data Catalogue at DOI: 10.17638/datacat.liverpool.ac.uk/2854. For readers wishing for a python implementation of the TSE, a suitable library can be found in scipy.stats.mstats.theilslopes.

## Supporting Information Available

1. Single-Molecule Conductance Measurements 1.1. Instrument details 1.2. Additional STM-BJ data 1.3. Additional flicker noise analysis data 1.4. Flicker noise measurements for full molecular data sets 2. Exponent Estimators, their Accuracy and Precision 2.1. Ordinary least-squares and the Theil-Sen estimator 2.2. Equivalence of OLS slope estimator of n and Pearson’s based minimization methods 2.3. Unbiasedness of the Theil-Sen estimator and it is fitting errors 2.4. Determination of the standard error for the Theil-Sen estimator 3. The sampling theorem applied to stochastic processes (PDF)
