# Supplementary material for: Digging through the (Statistical) Dirt: A Reproducible Method for Single-Molecule Flicker Noise Analysis
Source: J Phys Chem C Nanomater Interfaces. 2025 Feb 14;129(8):4097–104. doi: 10.1021/acs.jpcc.4c07780 (PMC11874016; doi:10.1021/acs.jpcc.4c07780)
Supplement: Supplementary file 1 — jp4c07780_si_001.pdf [file jp4c07780_si_001.pdf]

Digging Through the (Statistical) Dirt: A Reproducible Method for Single-Molecule Flicker Noise Analysis

James M. F. Morris,[a,b] Jarred Potter,[c] Demetris Bates,[a,#] Chuanli Wu,[a,+] Craig M. Robertson,[a] Simon J. Higgins,[a] Richard J. Nichols,[a] Paul J. Low[c] and Andrea Vezzoli[a,b,•]

a) Department of Chemistry, University of Liverpool, Crown Street, Liverpool L69 7ZD, United Kingdom
b) Stephenson Institute for Renewable Energy, Peach Street, Liverpool L69 7DF, United Kingdom
c) School of Molecular Sciences, University of Western Australia, Crawley, Western Australia 6009, Australia

- Corresponding authors: andrea.vezzoli@liverpool.ac.uk
  j.m.f.morris@liverpool.ac.uk

[#] Current address for Demetris Bates: School of Mathematical and Physical Sciences, University of Sheffield, Brook Hill, Sheffield S3 7HF, United Kingdom

[+] Current address for Chuanli Wu: Institute of Optoelectronic Materials and devices, Faculty of Materials Metallurgy and Chemistry, Jiangxi University of Science and Technology, Ganzhou 341000, China

1. Single-Molecule Conductance Measurements

1.1 Instrument details

We employed a modified Keysight 5500 STM in our studies. The instrument is equipped with a Femto DLPCA-200 transimpedance amplifier, operating at a 10^6 V/A gain. At his gain, the bandwidth of the amplifier (*i.e.* upper cutoff frequency -3 dB) is in excess of 200 kHz. We used an arbitrary waveform generator (Keysight 33522B) to impose a signal to the piezoelectric transducer responsible for moving the tip on the Z axis and to apply a DC bias to the molecular junction. All signals (piezo, bias, junction conductance) are recorded simultaneously by a National Instruments PXI system (PXIe-1062Q Chassis, PCIe-PXIe8381 interface, PXI-4464 DAQ). A $100\ \mathrm{k\Omega}$ resistor between the waveform generator and the STM tip prevents overload of the preamp when the conductance of the junction $G > 0.6\, G_0$. Data acquisition and partial processing is achieved on-the-fly through a bespoke LabVIEW Virtual Instrument (VI). The VI automatically separates the continuous data stream in individual fabricated junctions, and filters out data where a clean microcontact of $G \gg G_0$ could not be fabricated.

We used tips mechanically cut from a spool of Au wire (Goodfellows Precious Metals UK, 99.99+%) and substrates were prepared by evaporating ~ 120 nm Au (Advent Research Materials, 99.99+% shots) in a e-beam PVD system (Korvustech HEX) on freshly cleaved muscovite mica (Agar Scientific).

Details on further data analysis in the frequency domain is provided below.

1.2 Additional STM-BJ data

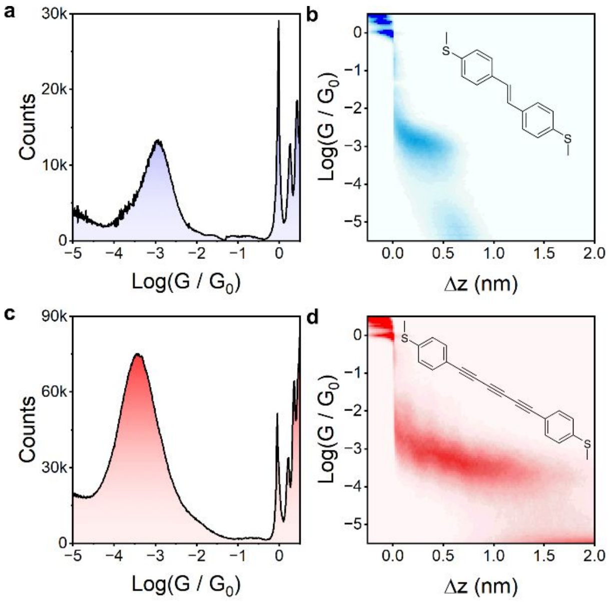

Figure S1: Single molecule conductance histograms of **(a)** molecule **1** and **(c)** molecule **2**. **(b)** and **(d)** the 2-dimentional conductance-displacement histograms for molecules **1** and **2** respectively. All data was collected at $200\ mV$ source-drain bias, in a $1\ mM$ solution in mesitylene, with a dataset size of 5841 for molecule **1** and 3956 for molecule **2**. Histograms and 2D plots compiled with $100\ bins/decade$ and $100\ bins/nm$.

1.3 Additional flicker noise analysis data

In addition to the main compounds studied, we also considered an oligophenylene ethynylene (OPE) molecule **3**. This molecule was chosen as it is a well characterised molecular wire but has known instability issues due to oscillatory interfacial interactions at the molecule-electrode interface. This instability results in an overestimated scaling exponent in the method without a stationarity requirement ($n_{TSE} = 1.46 \pm 0.04$, $n_{OLS} = 1.74 \pm 0.04$ – 1095 traces) when compared to the new method which includes the ADF step ($n_{TSE} = 1.28 \pm 0.05$, $n_{OLS} = 1.47 \pm 0.06$ - 594 traces). Due to this non-stationary nature we also see significantly reduced statistics after the data selection process for the newer method as a result of the ADF step in the processing procedure, indicting that a significant portion (44%) of the data was non-stationary. Overall, the newer method, as with molecule **2**, yields a scaling exponent indicating a stronger wavefunction overlap which is to be expected given the extended conjugation of the OPE class of molecules (Figure S2).

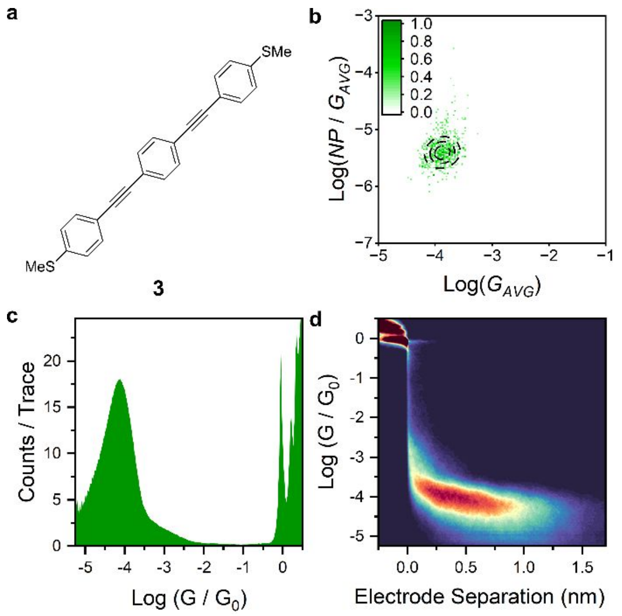

Figure S2: (a) Compound **3**. **(b)** Flicker noise analysis heatmap processed and analysed using the new method proposed (compiled from 594 traces). STM-BJ data for the **(c)** !-dimensional conductance histograms and **(d)** the 2-dimentional conductance-displacement heatmap, binned at $100\ bins/decade$ and $100\ bins/nm$ (compiled from 6131 traces).

1.4 Flicker noise measurements for full molecular datasets

A step-like voltage was applied to the piezo stack with each step consistent with the single-molecule junction extension as determined from the continuous STM-BJ measurements described in section S1.2, with each hold period lasting $100\ \mathrm{ms}$. Concurrently, the conductance was recorded in the same manner as the regular STM-BJ setup at acquisition rates of $100\ \mathrm{kHz}$ or $40\ \mathrm{kHz}$ for molecules **1** and **2** respectively.

Following this, the single-molecule conductance traces were fed into a bespoke LabVIEW program which used the derivative of the step function piezo signal to determine beginning and end of the junction. After isolation, the first datapoint of the traces were checked to ensure that they were below $0.5\ G_0$ and that the length of the junction was $100\ \mathrm{ms}$. These isolated traces were then processed further by removing the first and last $5\ \mathrm{ms}$ of the junction to reduce the impact of piezo creep effects. After which, the beginning and end ($5\ \%$) of the junction was compared with the conductance histogram of the linearly ramped STM-BJ

experiments. Traces with conductance values that fell within 2 standard deviations of the mean were retained for further analysis.

These junctions were then processed through an augmented Dickey-Fuller (ADF) unit root hypothesis test (adfuller(), statsmodels, Python 3.9, compiled 2022). If the p-values were below the assigned α-level ($\alpha = 0.05$) then the traces were considered sufficiently stationary for further processing. The traces that survived this process had their means (G_{AVG}) evaluated and subtracted (to avoid artificial changes to the power spectra towards $\sim 1/f^2$ type noise due to the non-zero mean of the finite period traces). The mean-subtracted, isolated, and stationary junctions were then processed by a power spectral density (PSD) algorithm (LabVIEW PSD.vi, LabVIEW 2019).

The PSD was then integrated between $100–1000\ Hz$ to avoid thermal and instrument noise sources to yield the noise power (NP). The NP was then compared with G_{AVG} after a Log-Log transform and the scaling exponent, n, and its error was determined from the slope using the Theil-Sen estimator (TSE) and its associated 67% confidence interval (see section S2.4 below for details). For transparency we have also included below (Table S1) the dataset sizes used for STM-BJ and flicker noise analysis both before and after the data selection procedure for all molecules.

Table S1: Dataset sizes for the STM-based experiments performed. Flicker noise analysis datasets are reported before and after data selection, using the selection criteria detailed in the manuscript.

Compound	STM-BJ<br># of traces	Flicker noise analysis Measurements<br># of traces selected / full dataset
**1**	5841	3125 / 8680
**2**	3956	1843 / 11141
**3**	6131	594 / 10845

2. Exponent Estimators, their Accuracy and Precision

2.1 Ordinary least squares and the Theil-Sen estimator

As discussed in the manuscript, $NP \propto G_{AVG}^n$ for a stationary molecular junction. Given that the only additive term to the noise will likely be due to the equipment (*e.g.,* amplifier noise) which is sufficiently small compared with the molecular junction noise, we can assume the equation takes the form:

$$NP = AG_{AVG}^n$$

Where A is a constant. As such we can linearise this equation to yield:

$$Log(NP) = nLog(G_{AVG}) + Log(A)$$

Whereupon determination of n amounts to determination of the slope between $log(NP)$ and $log(G_{AVG})$. If ordinary least squares (OLS) is used then the determined slope is that which minimises the absolute value

of Pearson's r (see S2.2 below for details). However, given that G_{AVG} is a random variable and long-tailed error distributions are probable in molecular junctions, such an estimator for the slope would be biased. This is because for the expected value of the slope estimator to equal the slope in the linear model, the independent variables are assumed to be fixed and the breakdown point of mean based estimators are 0 % (*i.e.* zero outliers must be present).[1,2] Also, OLS is only the best linear unbiased estimator (BLUE) when the error distribution is normal. Therefore, OLS is ideal in only a few cases. However, if a median based method such as the Theil-Sen estimator (TSE) is used, then such requirements are lifted, and the obtained slope is unbiased.[3]

In a comparable manner to the equivalence of the minimisation of $|r|$ and the OLS slope methods, the slope obtained from the TSE is that which minimises the absolute value of Kendall's correlation coefficient, t.[3] The latter is indeed known to be a more statistically robust measure of correlation than r, as t is non-parametric and less influenced by the presence of outliers or heavy tailed distributions.[2,4]

One major drawback of using the TSE to evaluate the slope is that the errors are more difficult to obtain than OLS.[1] However, this can be mitigated by using a percentile bootstrap or as is later discussed in section S2.4, can be determined by the number of traces and the appropriate z-score of the normal distribution. These methods greatly simplify the error analysis procedure.

2.2 Equivalence of OLS slope estimator of n and Pearson's based minimisation methods

As previously discussed, n can be determined by the minimisation of $|r|$ between $log(NP/G_{AVG}^{\lambda})$ and $log(G_{AVG})$. Where λ is an adjustable parameter, the value of which is taken to be the scaling exponent when $|r| = 0$. Throughout we have made reference to this being equivalent to the OLS slope between $log(NP)$ and $log(G_{AVG})$. In this section we will show this equivalence.

The expression $Log(NP/G_{AVG}^{\lambda})$ can be reformulated as:

$$Log(NP) - \lambda Log(G_{AVG})$$

And Pearson's correlation coefficient can be expressed as:

$$r = \frac{Cov(A,B)}{\sigma_A \sigma_B}$$

Where A and B are the values to be correlated. Using $Y = Log(NP)$, $X = Log(G_{AVG})$ and setting $r = 0$ the solution amounts to finding the conditions that satisfy

$$Cov(Y - \lambda X, X) = 0$$

For the same system, the linear regression model takes the form:

$$Y = \beta_1 X_i + \beta_0 + \varepsilon_i \quad (2.2.1)$$

Where β_1, β_0 and ε_i are the slope, y-intercept and error terms respectively. OLS assumes that ε_i and X_i are independent and that $\langle\varepsilon_i\rangle = 0$ as it is assumed that the intercept is centred at the mean of the errors. Expressing the covariance explicitly we find

$$\frac{1}{N-1}\sum_{i}^{N}((\bar{Y}-\lambda\bar{X})-(Y_i-\lambda X_i))(X_i-\bar{X})=0$$

Using equation 2.2.1 and after some rearrangement we find that

$$(\beta_1-\lambda)\left(\frac{1}{N-1}\sum_{i}^{N}(X_i-\bar{X})^2\right)-\left(\frac{1}{N-1}\sum_{i}^{N}\varepsilon_i(\bar{X}-X_i)\right)=0$$

From the assumptions of OLS, this can be simplified to

$$(\beta_1-\lambda)\sigma_x^2=0$$

Because $\sigma_x^2 \neq 0$ it must be that

$$\beta_1=\lambda$$

We note that this is true only when the assumptions of OLS hold and in the case of the double-log scale. As Pearson's correlation coefficient is only valid for linear datasets. Therefore, a Pearson's based approach on the non-linearised dataset can result in unreliable results and the assumptions of the OLS method must be met.

2.3 Unbiasedness of the Theil-Sen estimator and its fitting errors

The linear regression model is often expressed as:

$$Y_i=\beta_0+\beta_1 X_i+\varepsilon_i$$

Where ε_i is the error in Y_i not accounted for by X_i. With the slope given by the TSE:

$$\hat{\beta}_1=\underset{i<j\leq N}{\text{med}}\frac{Y_j-Y_i}{X_j-X_i}$$

These two equations yield:

$$\hat{\beta}_1=\underset{i<j\leq N}{\text{med}}\frac{(\beta_0+\beta_1X_j+\varepsilon_j)-(\beta_0+\beta_1X_i+\varepsilon_i)}{X_j-X_i}=\underset{i<j\leq N}{\text{med}}\frac{\beta_1(X_j-X_i)+(\varepsilon_j-\varepsilon_i)}{X_j-X_i}=\beta_1+\underset{i<j\leq N}{\text{med}}\frac{\varepsilon_j-\varepsilon_i}{X_j-X_i}$$

This is only unbiased if $\hat{\beta}_1-\beta_1=0$. It can be shown (theorem 5.1 in ref 3) that $\hat{\beta}_1-\beta_1$ is symmetric about zero given the error terms have a continuous distribution.[3,5] This followed from the symmetric nature of Kendall's correlation coefficient when $t=0$. Therefore, the TSE is an unbiased estimator of the slope.[3]

2.4 Determination of the standard error for the Theil-Sen estimator

The errors associated with the TSE can be obtained by bootstrapping through resampling with replacement of the NP and G_{AVG} datasets, and performing the TSE calculation.[1] 10,000 iterations of the bootstrap (B) were performed to investigate the distributional properties of the TSE for molecular junction datasets. It was found that the distribution of the slopes approximated a normal distribution up to the 99th percentile (Figure S3). However, it has been shown that if the regressors are random and the error distribution is absolutely continuous then the TSE is asymptotically normal.[3,6,7] Which would explain the approximate normality of the bootstrap results. If the fitting errors are continuous, it is reasonable to conclude that the distribution of the errors is absolutely continuous due to the nature of cumulative distribution functions. Thus, if NP and G_{AVG} are continuous random variables then the distribution of the TSE is asymptotically normal. Given the former is likely true, so too is the latter and the assumption of normality for a sufficient sample size is reasonable.

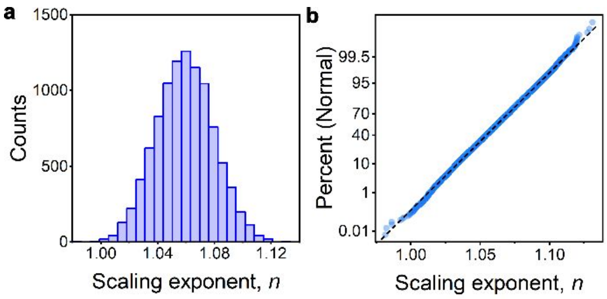

Figure S3: (a) The results of the 10,000 iteration bootstrap with replacement procedure for molecule **1**. **(b)** The probability plot for the resulting bootstrapped results (blue circles) compared to a normal distribution (dashed black line). The histogram was compiled at 156 bins/n. The sample mean and standard deviation of **(a)** were evaluated to be $\mu = 1.06$ and $\sigma = 0.02$, the same values were also found when a gaussian fitting was used instead.

As such, the upper and lower bounds for a given confidence interval (CI) of the TSE for $n > 10$ can be estimated through the standard error of numerator (difference sign score, Σ) in Kendall's correlation coefficient when $t = 0$.[3,8]

In other words, if Kendall's t is given by

$$t = \frac{2}{n(n-1)}\Sigma$$

Then we can find an index, k, where

$$k = z_{crit} \times SE_{\Sigma}(t = 0) = z_{crit}\sqrt{\frac{n(n-1)(2n+5)}{18}}$$

(2.4.1)

Where n is the number of traces and z_{crit} is the critical value of the associated α-level for the normal distribution. From equation 2.4.1 above, it is possible to obtain the indices of the upper $\left(\frac{1}{2}(N-k)\right)$ and lower $\left(\frac{1}{2}(N+k)+1\right)$ bounds for the set of ordered slopes given the total number of slopes, N. Finally, the CI can be determined from

$$CI_{100(1-\alpha)\%} = \left[\beta_{1,\frac{1}{2}(N-k)}, \beta_{1,\frac{1}{2}(N+k)+1}\right]$$

Given that both the bootstrapped value for the error (0.02) and the analytical value for the error using the above method (0.02) are in excellent agreement, the TSE error term is likely homoscedastic[1] and therefore, we suggest the use of the analytical value for its faster computation. A method for computation of the confidence intervals which includes tied values (highly unlikely in break-junction datasets at the quantities required to be problematic) can be found in the literature.[9]

3. The sampling theorem applied to stochastic processes

To illustrate why the observed sampling frequency needs to be larger than that which is predicted by the sampling theorem, we rederive the results of the theorem as applied to a stochastic process. Therefore, we begin with a sampled signal which can be expressed as:

$$X_S(t) = x(t)p(t)$$

Where $x(t)$ is the original time domain signal and

$$p(t) = \sum_{k=-\infty}^{\infty} \delta(t - kT_S)$$

is a delta function comb which samples the original signal k times with a period of T_S. The Fourier transform of $X_S(t)$ can be evaluated from the convolution of the transformed $x(t)$ and $p(t)$. *i.e.*

$$X_S(\omega) = X(\omega) * P(\omega)$$

Which can be expressed as

$$X_S(\omega) = \frac{1}{T_S} \sum_{k=-\infty}^{\infty} X(\omega) * \delta(\omega - k\omega_S)$$

where ω_S is the sampling frequency. Using the properties of the delta function we can obtain the final expression for the spectrum of the sampled signal:

$$X_S(\omega) = \frac{1}{T_S} \sum_{k=-\infty}^{\infty} X(\omega - k\omega_S)$$

Therefore, the frequency domain representation of the sampled signal is the Fourier transform of the true signal periodically repeated at k multiples of ω_S. The original signal can therefore be reconstructed given that B is less than half of the sampling frequency.[10] Thus, avoiding the overlap of two of the $k\omega_S$ centred spectra. This is the well-known sampling theorem and is often quoted as

$$\omega_S > 2B$$

However, for a stochastic process such as the conductance of a fixed displacement molecular junction, B is infinitely large and therefore, there will never be a ω_S for such a process that satisfies the sampling theorem formally. Nonetheless, given that the spectrum shows a $1/f$ dependence, we may conclude that for a sufficiently large ω_S the contributions from the neighbouring spectra will be negligible. Thus, despite no analytical threshold, Figure 2 in the main manuscript clearly shows the existence of an empirical one.

References

(1) Wilcox, R. A Note on the Theil-Sen Regression Estimator When the Regressor Is Random and the Error Term Is Heteroscedastic. *Biom. J.* **1998**, *40* (3), 261–268. https://doi.org/10.1002/(SICI)1521-4036(199807)40:3<261::AID-BIMJ261>3.0.CO;2-V.

(2) Croux, C.; Dehon, C. Influence Functions of the Spearman and Kendall Correlation Measures. *Stat. Methods Appl.* **2010**, *19* (4), 497–515. https://doi.org/10.1007/s10260-010-0142-z.

(3) Sen, P. K.; Estimates of the Regression Coefficient Based on Kendall's Tau. *J. Am. Stat. Assoc.* **1968**, *63* (324), 1379–1389. https://doi.org/10.1080/01621459.1968.10480934.

(4) Dietz, E. J. A Comparison of Robust Estimators in Simple Linear Regression. *Commun. Stat. - Simul. Comput.* **1987**, *16* (4), 1209–1227. https://doi.org/10.1080/03610918708812645.

(5) Wang, X.; Yu, Q. Unbiasedness of the Theil–Sen Estimator. *J. Nonparametric Stat.* **2005**, *17* (6), 685–695. https://doi.org/10.1080/10485250500039452.

(6) Wang, X. Asymptotics of the Theil–Sen Estimator in the Simple Linear Regression Model with a Random Covariate. *J. Nonparametric Stat.* **2005**, *17* (1), 107–120. https://doi.org/10.1080/10485250420002677743.

(7) Peng, H.; Wang, S.; Wang, X. Consistency and Asymptotic Distribution of the Theil–Sen Estimator. *J. Stat. Plan. Inference* **2008**, *138* (6), 1836–1850. https://doi.org/10.1016/j.jspi.2007.06.036.

(8) Kendall, M. G. A New Measure of Rank Correlation. *Biometrika* **1938**, *30* (1/2), 81–93.

(9) Fernandes, R.; G. Leblanc, S. Parametric (Modified Least Squares) and Non-Parametric (Theil–Sen) Linear Regressions for Predicting Biophysical Parameters in the Presence of Measurement Errors. *Remote Sens. Environ.* **2005**, *95* (3), 303–316. https://doi.org/10.1016/j.rse.2005.01.005.

(10) Shannon, C. E. Communication in the Presence of Noise. *Proc. IRE* **1949**, *37* (1), 10–21. https://doi.org/10.1109/JRPROC.1949.232969.
